# Supplementary material for: Are carbon emissions trading and green financial instruments synergistic? -Comprehensive quantitative research based on content analysis
Source: PLoS One. 2024 Mar 7;19(3):e0298601. doi: 10.1371/journal.pone.0298601 (PMC10919601; doi:10.1371/journal.pone.0298601)
Supplement: S1 Data — (ZIP) [file pone.0298601.s001.zip › Data/地级市Z_F工作报告.zip!/║┌┴·╜¡/╣■╢√▒⌡╩╨/└┤╫╘╣1⁄2╓┌║┼í╛┬φ┐╦╩2╛▌═°í┐_2016.docx]

各位代表：

现在，我代表市人民政府向大会报告工作，请予审议，并请市政协各位委员提出意见。

一、“十二五”时期简要回顾及2015年工作情况

“十二五”时期是我市发展极不平凡的五年。面对复杂的外部环境和繁重的改革发展稳定任务，我们在省委、省政府的正确领导下，按照市委的战略部署，紧紧依靠全市人民，认真贯彻落实党的十八大、十八届三中、四中、五中全会和习近平总书记系列重要讲话精神，坚持稳中求进工作总基调，主动适应经济发展新常态，改革创新、攻坚克难、砥砺奋进，全市经济社会发展迈上了新台阶。

——过去五年，经济运行保持在中高速增长的合理区间，经济综合实力显著增强。地区生产总值突破5000亿元大关，2015年预计达5750亿元，站上了新的历史起点。地方公共财政预算收入由238.1亿元增加到407.7亿元，年均增长11.4%，增速连续三年居东北四个副省级城市之首。累计完成社会固定资产投资20677亿元，实现消费品零售总额13651亿元，分别是“十一五”时期的2.7倍和2.1倍，内需对经济增长的拉动作用进一步提升。

——过去五年，产业项目建设实现了历史性跨越，产业转型升级取得显著成效。累计开工建设2000万元以上产业项目2671个，完成投资4702亿元，建成投产1744个，新增销售收入1900亿元以上、利税超过200亿元，华南城、万达文旅城等百亿元项目达到10个，凯斯纽荷兰、百事可乐等落户我市的世界500强企业已达38户，书写了哈尔滨产业项目建设史上的新篇章。农业综合生产能力大幅跃升，粮食总产量首次达到360亿斤，比2010年增加108亿斤，实现历史性“十二连增”。工业结构持续调整优化，哈飞福特汽车、中船重工燃气轮机等一批高端装备制造项目相继开工建设，食品工业产值由729亿元提高到1544.9亿元，成为我市首个超千亿元产业。新兴服务业发展势头强劲，金融业增加值占GDP比重由4.4%提高到6.4%，成为新的支柱产业，新设立创业投资引导资金成功撬动各类基金30亿元，在2003-2011年仅有2家企业上市的情况下，近四年来新上市、挂牌企业38家，金融支撑实体经济能力进一步增强；旅游总收入由381.5亿元提高到907.9亿元，冰雪节、迷人的哈尔滨之夏等系列品牌活动精彩纷呈，冰城夏都的知名度和美誉度进一步提升，旅游业发展进入历史最好时期。科技和产业加速融合，与哈工大、中科院等携手创建的6大平台累计转化重大科技成果1962个，全市高新技术企业发展到432家，科技引领、创新发展的热潮正在哈尔滨蓬勃兴起、涌动奔流。

——过去五年，城市面貌发生了日新月异的变化，城市功能品位显著提升。松北科技创新城、哈南工业新城、哈东现代物流产业带和哈西地区快速崛起，“一江居中、两岸繁荣”发展格局基本形成。累计投入2250多亿元，新建改造道路671条、桥涵156座，三环路基本实现贯通，地铁1号线载客运行，哈大、哈齐高铁相继开通，哈尔滨机场旅客吞吐量继2013年首次突破1000万人次后，去年攀升至1405万人次；建设各类源网厂站30座、排水泵站23处、地下管网6180公里，困扰市民多年的城市内涝、冬季供暖等难点问题得到有效解决，城市运行保障能力明显增强。重拳整治违建行为，打破背后利益链条，累计拆除违章建筑2.3万处、925.2万平方米，长期嚣张蔓延的违建之风得到基本遏制，顺应了民意，赢得了民心，维护了社会公平正义。持续提高城市精细化管理水平，主干街路环卫机械化作业实现全覆盖，主城区垃圾密闭运输率达到100%，违法小广告等城市顽疾得到有效治理。坚定实施绿色清冰雪，在确保冬季城市运行通畅和环境洁净的同时，更彰显了冰雪城市的独有魅力。成功战胜“布拉万”台风、特大洪水和暴雪等历史罕见的自然灾害，妥善处置城市供暖、道路交通和消防安全等方面突发事件，政府应急处置能力得到有效提升。加大水、气污染治理力度，累计撤并燃煤小锅炉2360台，延续20多年的“三沟”环境综合治理取得重大阶段性成果，不仅基本实现清水入河、结束污水直排松花江的历史，而且打造了新的城市生态景观。我市连续两年在中国最干净、最美丽城市排行榜上名列前茅。

——过去五年，改革开放不断向纵深推进，经济发展活力和动力显著增强。国资分类监管新体制初步形成，整合组建的交通、文旅、市政、住建等企业集团运营效益大幅提升，市属企业国有权益总额提高到“十一五”期末的2.6倍。率先在全省推进了农村土地确权登记颁证工作，全市提前三年完成了国家确定的改革任务，土地规模经营面积扩大到1790万亩，是确权前的3.7倍。率先在全国有立法权城市中取消了所有市级自设行政审批事项，建设项目审批时限平均压缩了50%以上，率先在全省推行了政府部门权责清单制度，实现了企业登记“四证合一、一照一码”，完成了市场监管、卫生计生、文新广电等“大部门”改革，率先在全省出台了行政问责、规范涉企执法行为、行政执法人员“六项禁令”等规定，累计查处失职渎职等破坏经济发展环境案件731起，问责公职人员1311人。简政放权进一步提高了行政效能、优化了营商环境，激发了民营企业活力，我市非公经济增加值占GDP比重由51.9%提高到55%。对俄合作中心城市建设扎实推进，先后开通了12条对俄客货运航线，哈欧货运班列实现常态化双向运营，对俄邮政小包裹发运量跃居全国首位。综合保税区已进入围网建设阶段，在白俄罗斯规划建设的中白工业园哈尔滨园为企业进入欧洲市场创造了条件。累计完成进出口总值245亿美元，是“十一五”时期的1.5倍，对欧美俄日韩等地区贸易额大幅提升，构建了全方位开放新格局。哈尔滨新区设立获国务院批复，成为全国唯一的以对俄合作为主题的国家级新区，为改革先行先试、产业集聚发展、扩大对外开放提供了载体支撑和新的动力引擎。

——过去五年，各项惠民承诺基本得到兑现，人民群众的幸福感和城市文明程度显著提高。城乡居民收入已分别达30977元和13375元，年均分别增长11%和13.7%，均高于同期GDP增速。累计完成惠民行动项目投资超过1500亿元，城乡居民就业、住房、社保和基本公共服务水平大幅提升，新增城镇就业64.5万人，开工建设各类保障房11.8万套，城乡低保标准由每人每月310元和每年1500元分别提高到550元和3500元，上学难、看病贵等问题得到有效缓解。全心全意为群众办实事、解难事，老旧小区基本实现物业全覆盖，历史遗留的8.7万户居民房屋产权证办理问题得到了解决，长期在外临时居住的6万户棚改回迁居民喜迁新居，道外桥头屯和新一地区的1.3万户内涝居民全部得到妥善安置，越来越多的困难群众圆了“安居梦”。精神文明、民主法治和平安哈尔滨建设扎实推进，构建新型社会治安立体防控体系和创建最具安全感省会城市取得明显成效，刑事案件万人发案率连续五年保持省会城市最低，人大代表建议、政协提案办复率始终保持100%，我市先后被评为全国社会治安综合治理优秀城市、和谐社区建设示范城市。经过三年创城攻坚，哈尔滨成功跨入全国文明城市行列，市民自豪感和城市吸引力明显增强，为加快发展注入了强大的精神动力。

各位代表，2015年既是“十二五”收官之年，也是我们应对经济下行压力，不断夯实打牢科学发展基础的重要一年。我们按照市委十三届七次、八次全会和市十四届人大四次会议部署，咬定青山不放松，一以贯之抓落实，重点推进了以下工作：

（一）不断加大产业项目建设力度，进一步增强经济发展的内生动力。牢牢把握产业发展这一稳增长、调结构的核心，坚持规模速度与质量效益并重，多措并举、精准发力，开创了产业发展提速增效新局面。一是持续壮大产业规模。全年新开复工2000万元以上产业项目1030个，其中新开工531个，完成投资900亿元。新签约产业项目525个，实际使用内资1998.3亿元、外资29.9亿美元，分别比上年增长13.9%和10%。二是持续推动产业升级。重点推进的通联新能源客车、万鑫石墨园等200个工业项目和107个服务业项目进展顺利，哈飞通用直升机、航天海鹰3D打印等50个高端制造业项目建成投产，永泰城、枫叶小镇奥特莱斯、凯宾斯基酒店等大型服务业项目开业运营，有力地提升了我市产业发展层次和水平。三是持续实施创新驱动战略。加快推进国家首批“小微企业创业创新基地”示范城市建设，启动实施科技创业三年行动计划，新增科技型中小微企业840户、销售收入超过500万元的171户。中国农科院主食加工技术研究院建成投用，哈工大焊接集团、哈船动力等高新技术企业成功运营，开辟了科技成果资本化产业化新途径。四是持续集聚产业发展要素。哈尔滨股权交易中心投入使用，威帝电子、奥瑞德、中飞新技术成功上市，利用资本市场助推产业发展能力进一步增强。园区建设引导资金扩大到8亿元，重点园区承载功能持续提升，经开区、高新区、利民和宾西开发区呈现出产业集聚、产城融合发展新态势。通过全市上下的不懈努力，产业规模持续扩大，产业结构不断优化，为我市经济长远发展奠定了更加坚实的基础。

（二）深入实施富民强县战略，进一步壮大县域经济整体实力。全面落实国家两大平原现代农业综合配套改革任务，持续加大“三农”支持力度，县域经济发展潜能加快释放。一是大力实施产业强县工程。围绕发展特色县域经济，新建续建亿元以上产业项目146个，完成投资120亿元，奥瑞德四期、东方农业科技园等70个项目建成投产，巴彦工业园晋升为省级开发区，县域规模以上工业总产值占全市比重达34%。二是加快转变农业生产方式。着力提高机械化、科技化、集约化水平，农业综合机械化率提高到94%，现代农业示范园区（带）核心区面积扩大到1556万亩，绿色、有机农产品认证面积增加到1282万亩，国家地理标志产品达到26个，新建扩建规模化养殖场（区）135个，五常大米保真打假整治行动成效显著，农产品质量监管能力进一步提升。率先在全省启动了方正整县、平房整区集体产权制度改革试点，以及双城、五常新型社会化服务试点，农村产权流转交易平台、土地纠纷仲裁机构实现全覆盖，各类新型农业经营主体发展到14668个，土地规模经营基础进一步夯实。三是扎实推进新型城镇化建设。撤销乡镇7个、新设街道办事处10个，建设美丽乡村示范村100个，新建农村饮水安全工程673处，68个贫困村实现整村脱贫，农村公路交（竣）工通车450公里，农村面貌持续改观，城乡一体化发展步伐进一步加快。

（三）持续强化城市规划建设管理，进一步提升城市承载力和吸引力。围绕建设现代化国际化城市目标，以创建全国文明城市为牵动，推动城市建设发展向更高层次迈进。一是城市基础设施建设统筹推进。着力加强城市规划的科学性和权威性，推动基础设施建设向城市新区、产业园区延伸拓展，华南城、综保区等配套路网体系基本形成，滨北公铁两用桥、一面街地道桥等涉铁配套工程顺利推进，地铁1号线三期、2号线、3号线启动建设，国家地下综合管廊试点进展顺利，新建续建停车场25处，新增更新公交车辆600台，“公交都市”创建走在全国前列。二是城市精细化管理水平稳步提高。持续加大牌匾广告、市场摊区、交通“三乱”等专项整治力度，全年创建环境卫生达标街巷、庭院200个，取缔违规牌匾广告1万余处，规范市场摊区155个，查处交通违法行为462万件，城市面貌实现更大改观。三是生态环境治理力度持续加大。重点实施污染减排项目204个，拆改整治10吨以下燃煤锅炉594台，达标治理10吨以上燃煤锅炉328台，淘汰黄标车及老旧车6.02万辆，超额完成年计划43.3%，新建万吨级生物质秸秆综合利用项目15个，建成团结镇等4个污水处理厂，城市污水集中处理率、集中供热普及率分别提高到90%和87.2%，城市空气优良天数达到227天，优秀天数比上年增加24天。城区新植树木21.6万株、新增绿地67.4公顷。哈尔滨这座城市变得越来越洁净、越来越有序、越来越秀美、越来越有魅力。

（四）全面深化改革扩大开放，进一步激发经济社会发展活力。坚持向改革要动力，以开放拓空间，加快构建统一开放、竞争有序的市场新机制。一是深入推进简政放权。政府事权精简了52.5%，与行政审批相关中介服务事项减少了35%，商事制度改革充分激发了全民创业创新活力，新注册内资企业23668户、外资企业170户，分别增长16.4%和34.9%。二是稳步推进国企改革。市政与建工集团合并运营，哈轴制造公司、马迭尔集团食品板块成功实现合资重组。通过政企分开、资源整合、实施成本规制等有效措施，解决了公交企业管理粗放、效益偏低等历史遗留问题。市物业供热集团14.8亿元债券成功发行，成为我市首个国企资产证券化项目。三是切实加强财税管理。重点建立了中期财政规划管理和跨年度预算平衡机制，政府到期债务全部偿还，进一步完善了市对区县（市）财政管理体制。大力推广政府与社会资本合作，地下综合管廊、地铁2号线、3号线二期等6个项目成功引入社会资本达468亿元。加强税收征管挖潜，强化财政预算支出管理，全年财政支出达824.8亿元，为全市经济社会发展提供了有力保障。四是继续扩大对外开放。哈尔滨机场实行72小时过境免签，成功开通东北首条第五航权航线，哈俄韩陆海联运通道正式启用，哈尔滨铁路集装箱中心站建成投用，龙运物流、瀚润供应链等11家“两头在外”企业签约入驻综保区，落户临空经济区的大飞机拆解、绿地空港城、跨境电商物流基地等项目建设进展顺利，我市首个对俄跨境海外仓在叶卡开工建设，成功举办第二届中俄博览会暨第26届哈洽会、首届中俄文化艺术交流周和第七届韩国首尔哈尔滨周，对外开放的深度和广度不断拓展，我市经济活跃度和对外影响力得到了新提升。

（五）着力保障和改善民生，进一步为百姓办实事办好事解难事。面对持续加大的财政减收增支压力，我们集中财力保民生，市财政用于民生领域支出达577.4亿元，各项民生任务全面落实。一是不断改善居民居住条件。开工建设各类保障房14210套、竣工16523套，改造农村泥草房5万户，新增物业管理面积606万平方米，维修老旧住宅734万平方米，创建市级以上物业管理示范小区106个。二是不断提高社会保障水平。企业退休人员基本养老金提高到人均每月2083元，为51.1万困难群众发放救助金16.9亿元，农村日间照料中心和社区养老服务站基本实现全覆盖。城镇居民医疗保险人均补助标准提高到380元，新农合统筹区域内政策性报销比例超过80%、人均筹资标准提高到470元。三是不断加强基本公共服务。创建义务教育标准化学校50所、素质教育特色（示范）学校26所，新建改造中小学校36所。组建医疗联合体42个，政府办基层医疗卫生机构全部实行药品零差率销售。城区健身设施和城乡公共文化服务网络实现全覆盖，哈尔滨大剧院、国际油画展示交易中心、“731”遗址新馆建成开放，纪念抗战胜利70周年系列活动成功举办。民族宗教、外事侨务、双拥和预备役、统计、审计、信访、气象、人防、地震、档案、地方志、残疾人等工作取得新进步。

五年来的发展实践使我们深切地感到，面对新常态下的各种压力和挑战，之所以能够实现经济社会平稳健康发展，关键在于我们始终坚持正确发展方向，牢牢把握中央“四个全面”战略布局和稳中求进总基调不动摇，深入贯彻落实省委、省政府和市委战略部署不懈怠，全市各级领导班子团结奋进，人大代表、政协委员鼎力支持，凝聚了万众一心干事创业的强大合力；关键在于我们始终坚持发展第一要务，以功成不必在我的胸怀和持之以恒的韧劲，举全市之力抓产业上项目、调结构转方式、打基础攒后劲，积蓄了加快发展、跨越赶超的新动能；关键在于我们始终坚持敢于负责、敢于担当、敢于碰硬，直面问题不回避、攻坚克难不退缩，在深化行政管理、国资国企、资源配置市场化等重点领域改革中，勇于破除体制机制弊端，从根本上解决多年积累的深层次矛盾，充分激发了经济发展的内生动力和社会创造力；关键在于我们始终坚持党的群众路线，把保障改善民生作为政府工作的出发点和落脚点，想群众之所想、急群众之所急，千方百计破解民生难题，努力让改革发展成果更多更公平地惠及百姓，增强了广大群众投身城市建设发展的凝聚力和向心力；关键在于我们始终坚持科学执政、依法行政、从严治政，正确处理政府和市场的关系，充分践行政府调控职能，全面加强公务人员队伍作风建设，提高了行政效能和服务水平，优化了营商环境，极大地提升了区域经济发展的吸引力和竞争力。五年积累的经验弥足珍贵，取得的成绩来之不易。在此，我代表市人民政府，向所有关心支持哈尔滨事业发展的同志们、朋友们，向广大驻哈解放军指战员、武警官兵，致以崇高的敬意和衷心的感谢！

在肯定成绩的同时，我们也清醒地认识到，前进中仍面临许多困难和挑战：稳增长促发展的任务仍很艰巨，部分企业订单下降、开工不足、效益下滑的状况仍在持续，产业项目招商竞争更加激烈，化解地方债务、去房地产库存压力进一步增大；产业整体实力不强、规模不大、结构不优等状况还没有根本改变，产业发展面临着传统优势产业竞争力减弱和战略性新兴产业发育不充分的双重压力，加快产业转型升级、提质增效仍然任重道远；市场化进程不快，长期计划经济形成的思维定式和路径依赖依然存在，创新创业不足，公共资源配置市场化程度不高，混合所有制改革还比较滞后，民营经济和开放型经济发展水平相对偏低；公共服务和民生保障能力与群众期待还有差距，交通、安全、环保、物业管理等方面涉及群众切身利益的现实问题仍需努力加以解决，脱贫攻坚任务仍很繁重；政府自身建设尚需加强，部分公务人员专业化水平不高，责任担当意识不强，不作为、乱作为、不会作为等现象仍然存在，公共权力部门化、部门权力利益化等问题尚未根除，损害群众利益和腐败行为仍时有发生。对此，我们要坚持用发展的办法解决前进中的问题，不断推动经济社会持续健康发展。

二、“十三五”时期发展总体思路和工作的基本把握

“十三五”时期是全面建成小康社会的决胜期，也是哈尔滨全面振兴发展滚石上山、爬坡过坎的攻坚期。从不利因素看，当前世界经济曲折复苏，市场需求不振的状况短期内难以改观，哈尔滨与全国全省一样面临着经济换挡减速、结构调整阵痛、新旧动力转换的较大压力。从有利条件看，新一轮东北老工业基地振兴、龙江丝路带建设、哈长城市群和哈尔滨新区等国家战略深入实施，为我们深化改革、扩大开放、加速转型升级创造了难得的历史性机遇。特别是经过几年来的不懈努力，我们推动发展的要素充分集聚，改革红利和发展潜能加速释放，实现哈尔滨加快发展的产业基础更加坚实、体制机制更加完善、干事创业的热情更加高涨。可以说，“十三五”时期，发展机遇与挑战并存，困难中孕育更大希望。只要我们坚定发展信心，保持战略定力，进一步增强机遇意识和危机意识，奋发图强、埋头苦干，哈尔滨就一定会在全面建成小康社会的征程中谱写出更加绚丽美好的新篇章！

今后五年政府工作的指导思想是：高举中国特色社会主义伟大旗帜，全面贯彻党的十八大和十八届三中、四中、五中全会精神，以马克思列宁主义、毛泽东思想、邓小平理论、“三个代表”重要思想和科学发展观为指导，深入贯彻习近平总书记系列重要讲话精神，按照“五位一体”总体布局和“四个全面”战略布局的要求，牢固树立和贯彻落实创新、协调、绿色、开放、共享的发展理念，适应经济发展新常态，落实省委十一届六次全会和市委十三届九次全会部署，坚持改革开放，坚持稳中求进工作总基调，以提高发展质量和效益为中心，着力完善体制机制，着力推进结构调整，着力鼓励创新创业，着力保障和改善民生，统筹推进经济、政治、文化、社会、生态文明建设，加快老工业基地振兴，确保如期全面建成小康社会，为建设现代化国际化城市奠定坚实基础。综合考虑各方面情况，预期“十三五”时期地区生产总值年均增长6%-6.5%，城乡居民收入增长与经济增长同步。为实现上述思路目标，我们必须深刻分析和把握哈尔滨发展的阶段性特征和规律性要求，着重在贯彻落实五大发展理念上下功夫、求突破。

——坚持创新发展，切实在发展动能转换中提质量、增效益。创新是引领发展的第一动力，创新不足也是新常态下制约我市加快发展的主要症结。必须把创新放在全市经济发展全局的核心位置，加快构建以创新为主要支撑的经济体系和发展模式，按照市场主导、企业主体、政府推动的原则，从供需两端加大结构性改革力度，以中国制造2025哈尔滨行动计划为牵动，重点实施总投资超过8000亿元的900个重大产业项目，着力在工业强基带动产业振兴、科技创新引领“双创”发展、创新体制机制打造便捷高效营商环境等方面实现新突破。力争到2020年，食品工业总产值突破3000亿元，高端装备制造、文化旅游、以新一代信息技术为代表的新兴产业分别跨上千亿级台阶，科技进步贡献率提高到65%，基本形成以现代农业为基础、先进制造业与现代服务业双轮驱动的现代产业体系，建立完善同市场完全对接、充满内在活力的体制机制，努力使今后五年成为内生动力不断增强、发展效益和质量得到更大提升的五年。

——坚持协调发展，切实在全面提升发展整体性上拓空间、增后劲。协调是持续健康发展的内在要求，哈尔滨作为后发城市，城乡发展不平衡、经济和社会发展不协调的问题仍比较突出。必须坚持区域协同、城乡一体、物质文明和精神文明并重，突出抓好总投资超过6000亿元的400个重大城乡基础设施项目建设，不断完善江南主城区城市功能，着力提升城市新兴发展区域的产业和人口集聚能力，加快建设双城、阿城、呼兰、宾西四个卫星城和五常、尚志、方正—通河区域次中心城市，积极推进巴木通方依区域经济一体化发展，持续推动城乡公共服务均等化、公共资源均衡配置，进一步提高城乡居民文明素质，构筑区域协调的城乡发展新格局。力争到2020年，哈长城市群和哈尔滨都市圈初具规模，覆盖城乡的基础设施和公共服务体系进一步完善,户籍人口城镇化率提高到55%，全国文明城市创建成果得到巩固提升，努力使今后五年成为城乡发展更趋协调、精神文明与和谐社会建设取得更大成效的五年。

——坚持绿色发展，切实在建设美丽宜居哈尔滨上求突破、见实效。绿色是永续发展的必要条件和人民对美好生活追求的重要体现，我市江河、湿地、森林等生态资源优良，打造美丽哈尔滨我们有基础、有条件，更有巨大的发展潜力。必须坚持绿色富市、绿色惠民，重点在培育绿色经济、倡导绿色生活、加强环境保护和污染治理等方面实施一批重大项目，在全社会加快形成绿色发展方式和生活方式。力争到2020年，健康养老、节能环保等新兴绿色产业发展取得重大进展，新能源汽车、建筑节能、低碳消费成为绿色生活新风尚，水污染、雾霾等损害群众健康的突出问题得到有效解决，秸秆综合利用率提高到85%，城市主城区全部淘汰20吨及以下燃煤小锅炉，重污染天气比“十二五”期末减少60%，松花江和“三沟一河”水生态保护与修复任务全面完成，哈尔滨群力外滩生态湿地公园等重点区域建成向市民开放，松花江生态景观带、文化旅游带、休闲经济带基本建成，让天蓝地绿水净的良好生态成为最普惠的民生福祉，努力使今后五年成为生态更优良、环境更宜居、生活更健康的五年。

——坚持开放发展，切实在打造全方位对外开放新格局上强基础、扩优势。开放是繁荣发展的必由之路，也是把哈尔滨沿边开放区位优势转化为经济优势的必然选择。必须积极抢抓国家“一带一路”、龙江丝路带和哈尔滨新区建设战略机遇，以打造对俄合作中心城市为牵动，不断完善开放功能、拓展合作空间，重点在物流通道建设、搭建产业合作平台、对外贸易提档升级、推动多元化国际合作等方面迈出更大步伐。力争到2020年，全面打通哈尔滨至欧洲、北美、日韩和港澳台地区贸易通道，综保区、临空经济区对外产业承接的载体功能更加强化，哈尔滨作为国家沿边开发开放重要经贸平台、物流枢纽、外向型高端制造业基地和全方位交流合作中心的作用更加凸显，努力使今后五年成为开放程度明显提高、发展活力持续增强、开放型经济发展水平全面提升的五年。

——坚持共享发展，切实在保障和改善民生上补短板、增福祉。共享是中国特色社会主义的本质要求。必须正确处理好经济发展与改善民生的关系，按照人人参与、人人尽力、人人享有的要求，坚守底线、突出重点、完善制度、引导预期，更加注重机会公平，不断增进民生福祉，以惠民行动为抓手，着重在增加公共服务供给、实施脱贫攻坚工程、促进就业创业、完善社保和住房保障体系等方面持续加大力度。力争到2020年，城镇新增就业55万人，城乡低保标准和企业退休人员基本养老保险待遇稳步提高，基本完成城镇棚户区、城中村和危房改造，延寿、木兰、巴彦3个贫困县全部摘帽，163个贫困村、20.1万贫困人口全面脱贫，市奥体中心等重点文体设施建成投用，教育、卫生、医疗、养老等公共服务水平大幅提升，努力使今后五年成为全市人民幸福指数明显提高、共享改革发展成果、得到实惠更多的五年。

三、2016年主要任务

2016年是“十三五”规划的开局之年，做好今年工作意义特别重大。按照市委十三届九次全会要求，我们要深入贯彻落实省委、省政府实施“五大规划”和十大重点产业建设战略部署，聚焦全面建成小康社会，着力加强结构性改革，全力做好稳增长、促改革、调结构、惠民生等重点工作，精准发力、扎实起步，努力实现“十三五”经济社会发展的良好开局。

今年主要预期目标是：地区生产总值增长6.5%-7%；固定资产投资增长10%左右；规模以上工业增加值增长3%以上；社会消费品零售总额增长10%左右；地方公共财政预算收入增长2%以上；市属外贸进出口总额增长5%左右；城镇居民人均可支配收入增长7%以上，农村居民人均可支配收入增长8%以上；城镇居民登记失业率控制在4.4%以内。为实现上述目标，我们要着重做好以下八项工作：

（一）坚定不移抓产业上项目，着力积蓄稳增长、调结构、促转型的新动能。牢牢扭住产业建设这一牵动经济发展全局的“牛鼻子”，以供给侧改革带动产业结构调整，全力以赴上项目扩增量、促升级增动力，重点抓好100个投资5000万元以上工业项目和100个5亿元以上园区基础设施及服务业等重大项目，切实增强经济稳增长的产业支撑力和带动力。一是加快工业经济转型升级步伐。以做大做强千亿级产业为重点，不断提升全市工业的整体实力，确保规模以上工业增加值超千亿元。依托农产品质量和“寒地黑土”品牌优势，加快推进雀巢奶粉、宾西牛业等项目建设，进一步巩固提升千亿级食品产业板块。以实施智能制造工程为牵动，支持重点企业加大技改力度，加快推动制造业向中高端迈进，突出抓好哈南机器人、东安铝镁合金铸造等30个亿元以上项目建设，推动中船重工燃气轮机、通联新能源客车等项目建成投产，确保哈飞福特汽车顺利下线，实现装备制造业产值突破千亿元。重点瞄准生物医药、新材料、节能环保、新一代信息技术等前沿领域，积极引入战略投资者，加快推进乐泰药业、万鑫石墨、阿里巴巴哈尔滨平台、朗格斯特寒地节能产业基地等项目建设，努力打造千亿级新兴产业板块。二是促进服务业提档升级。继续推动金融创新发展，加快推进哈尔滨创新型金融示范区等功能区建设，充分发挥创业投资引导资金作用，大力发展天使、创业、产业等投资基金，支持企业依托多层次资本市场发行股票、债券、资产证券化等融资工具，不断提高资本市场直接融资能力。加快发展现代物流业，重点抓好华南城对俄经贸物流园、传化智能公路港等项目建设，进一步完善城市共同配送体系。不断提升会展经济、总部经济发展水平，重点抓好国际农博中心、HBP企业总部基地等项目建设，高质量办好农博会、寒博会等品牌展会。认真落实国家去库存各项政策措施，促进房地产业持续发展。借助我市生态优势和医疗资源，推进养生度假、健康和候鸟式养老等业态发展，支持各类市场主体开发养老服务和产品。积极推动旅游文化时尚产业融合发展，依托冰雪优势、音乐名城、欧陆风情等特色旅游资源，加强顶层设计，进一步健全“吃住行游购娱”一体化服务体系，突出抓好万达文旅城、波塞冬海洋王国、五洲动漫城、香港卫视东北亚总部等项目建设，完善旅游配套设施功能，A级旅游景区的水冲式公厕达到200个以上，设计推出一批具备鲜明文化特征和时尚要素的旅游产品，重点办好贯穿旅游季、特色鲜明的系列驻场演出，持续提升冰雪节、迷人的哈尔滨之夏等系列活动的国际影响力，全年实现旅游总收入增长14%以上，成为我市又一个千亿级产业。三是加大招商引资力度。全力开展多领域、专业化、精准式招商，进一步做好签约项目和央企合作项目的推进落实工作，更多引进开工一批投资规模大、创税能力强、产业带动作用突出的大项目、好项目。着力打造优良的营商环境，深入开展降低实体经济企业成本行动，全面落实国家减税降费等优惠政策，促进企业提质增效。力争全年引进亿元以上项目200个，实际使用外资、内资分别增长7%和10%左右。四是强化园区载体作用。把重点产业项目扶持资金提高到25亿元，着力推动主导产业发展和重点园区建设，吸引更多的新技术、新产业向园区集聚。经开区围绕打造高端装备、食品、信息三大产业集群，重点加快50个产业项目建设，确保可口可乐搬迁扩产等20个项目建成投产。高新区突出抓好清华万博等10个专业化园区建设，集聚中小微科技企业超过1500家。利民开发区着力推进元亨动物疫苗等48个项目建设，确保建成投产10个以上。宾西开发区重点发展包装、食品等产业,确保鑫荷包装等5个项目竣工投产。力争通河、延寿工业园晋升为省级开发区。

（二）加快建设创业创新型城市，着力打造促进经济转型发展的新引擎。坚持把发展的基点放在创新上，加快推动科技与经济深度融合、创新与创业相互促进，增创区域竞争新优势。一是加强科技创新引领。重点在特种作业机器人、新一代信息技术等领域实施一批重大攻关项目，解决好核心技术问题，在引领智能制造、互联网与制造业融合等科技研发上取得新进展。以哈工大市校科技合作第二轮试点为牵动，探索建立科技成果转化股权激励机制，推广以项目团队为主体，政府引导、校所参与、社会资本跟进的科技成果转化新模式，力争全年科技成果落地转化300项。二是强化创新要素保障。加快建立丝路科技合作基地战略联盟，组建哈尔滨国际科技合作联合创新中心，吸引集聚更多科技研发和技术转移机构入驻。完善科技创新融资模式，加强与资本市场合作，进一步扩大科技风险投资规模，全面实施企业先行投入、市场评价效果、政府事后补贴的科技创新创业券计划，建立起激励加约束的创新扶持新机制。充分用好1亿元且上不封顶的人才引进资金，吸引更多高层次紧缺实用人才集聚，为创新发展提供智力支撑。三是拓展创业创新空间。不断完善科技企业孵化体系，重点抓好哈以、哈南生物医药、龙门科技等孵化器建设，推广创业社区、创客空间、创新工场等新型孵化模式，力争新增在孵企业300家以上。深入推进国家小微企业创业创新基地示范城市建设，制定实施2020“互联网+”哈尔滨行动方案，率先在“互联网+”精准农业、先进制造、益民服务等领域推出一批示范项目，不断加强城市公共免费无线网络等基础设施和服务平台建设，全年新增小微企业1.6万户，营业收入增长20%以上。四是推动创业带动就业。坚持就业优先战略，支持灵活就业，发展新就业形态，通过小额贷款担保、创业项目和场租补贴等有效措施，推动大学生、科技人员和农民三支队伍创新创业。全年新增城镇就业10.3万人，转移农村劳动力超过150万人。

（三）统筹推进城乡一体化科学发展，着力释放县域经济发展潜能。按照两大平原现代农业综合配套改革部署，更加注重一二三产业融合，进一步促进农业增效、农民增收、农村繁荣。一是加快推进农业生产方式转变。突出市场导向，推动种植结构优化调整，启动建设20个现代生态农业高标准示范园区，加快打造沿江优质稻米产业带和五常水稻、双城玉米等有机生产基地，粮食总产量稳定在320亿斤左右，农产品质量和效益持续提升，努力使农业生产实现“种得好、卖得好、种得更好”的良性循环。大力发展现代畜牧业，新建扩建规模化养殖场（区）30个，肉蛋奶总产量保持在300万吨以上。不断提高农业水利化、机械化水平，重点推进通河二甲沟水库等水利工程建设，新建大型农机专业合作社15个，努力夯实现代农业发展基础。二是深入推进产业强县工程。把资源精深加工作为县域工业发展的主攻方向，加快构建以绿色食品为重点、各具特色的农业产业化体系。突出抓好巴木通方依一体化特色产业带建设，加快推进巴彦皮草产业基地、木兰颐康元主食加工等项目，力争县域工业总产值占全市工业比重提高到35%以上。三是深化农村综合改革。巩固扩大农村土地确权登记颁证成果，实现四级农村产权交易平台互联互通。积极培育新型农业经营主体，推动合作社由生产主体向全方位经营主体转变，鼓励工商资本发展适合企业化经营的现代种养业、农产品加工流通和农业社会化服务，全年新增市级以上农民合作规范社100个、农业产业化龙头企业178家。扎实推进农村集体产权制度改革试点，年内基本完成31个试点村的改革任务。四是创新完善农业社会化服务体系。启动市、县和新型农业主体三位一体科技服务试点工作，大力培养新型职业农民，重点推广农民合作社资金互助和农业投保入股新模式，集中打造市级统筹政策性农牧业融资担保服务平台，大力发展“互联网+营销”，使优质农产品卖出好价钱，让农民获得更大收益。五是扎实推进新型城镇化和美丽乡村建设。深入开展国家新型城镇化试点，筹建新型城镇化发展基金，综合改造10个国家和省重点镇。积极推进农村集体建设用地入市和一元化户籍制度改革，有序推进农业转移人口市民化。大力开展美丽乡村创建活动，打造哈五、哈双等6条示范带，启动建设示范村100个，集中治理自然屯1000个，实施农村饮水安全巩固提升工程130处。突出抓好农村交通体系建设，新建农村公路300公里，交工通车200公里，改造危桥80座，确保木兰松花江公路大桥建成通车，为农业农村发展创造更加有利的基础条件。

（四）加快推进现代化国际化城市建设，着力打造绿色生态宜居家园。进一步提高城市集约高效发展水平，努力让群众在城市生活得更便捷、更舒心、更美好。一是完善城市规划。着手修编城市总体规划（2016-2030年），科学编制城市综合交通、海绵城市等专项规划，精心做好松花江沿线生态景观等重要节点城市设计，为彰显城市风格魅力提供引领和支撑。二是提升城市功能。进一步疏解城市交通堵点，畅通区域微循环，同步推进地铁1号线三期、2号线一期和3号线一期、二期工程建设。配合铁路航空部门，全力推进滨北公铁两用桥、哈站枢纽改造、哈佳、哈牡客专、哈尔滨机场扩建等工程建设。加快实施红旗大街等地下综合管廊试点项目，完善智慧管网信息系统，持续提高城市运行保障能力。积极推进松花江水源供水工程建设和江南主城区二次供水设施改造，努力保证城市供水安全。新增更新公交车辆600台，启动建设公交首末站4座、枢纽站1座，不断提升“公交都市”创建水平。三是强化城市管理。坚持精细化、数字化管理，加大对背街背巷、居民庭院、街路餐饮、城乡结合部等重点区域环境治理力度，切实提高环卫机械化作业水平。重点推进向阳垃圾场焚烧发电、松北呼兰垃圾场等项目建设，加快构建科学规范的垃圾处理体系。始终保持拆违控违高压态势，从严整治乱停乱放、乱贴乱画等城市顽疾，确保城市面貌和环境秩序实现更大改观。四是改善城市生态环境。深入落实大气污染防治行动计划，持续加大燃煤质量管控和分散燃煤小锅炉撤并力度，确保燃煤电站和热电联产机组稳定达标排放，全部淘汰剩余黄标车，秸秆综合利用率达到63%。扎实开展水污染防治行动计划，巩固扩大松花江干流和城市内河治理成果，确保松花江断面水质保持或优于功能区划标准。启动建设松花江群力外滩生态湿地公园，加快建设中东铁路公园，着力打造生态健康休闲宜居集中区。全年人工造林4.6万亩，城区新植树木25万株、新增绿地66公顷，努力使人民群众的生活环境更加宜居健康，让哈尔滨的天更蓝、地更绿、水更清。

（五）坚决破除一切制约科学发展的体制机制弊端，着力推动重要领域改革取得更大进展。突出问题导向，注重精准发力，强化完善制度，狠抓推进落实，确保改革不断见到成效。一是扎实推进政府职能转变。继续做好审批事项“接放管”，重点建立部门权责清单动态调整、责任追究长效机制，更好发挥网上审批作用，优化审批流程，更多运用大数据、物联网、随机抽检等方式加强事中事后监管，切实把简政放权、放管结合、优化服务的要求落到实处。全面深化部门内设机构“大处室”改革，从根本上解决多头管理、相互掣肘、效率低下等问题。持续推进事业单位分类改革，衔接好有关社保政策，完成与审批相关的中介服务机构“事转企”改革任务。二是继续深化国资国企改革。坚持以管资本为主，完善国资管理体制，加快推进哈投、工投集团改组为国有资本投资运营公司，积极支持哈电与一重、中核集团战略合作。分类实施国企改革，通过股权多元化等方式，提高哈药、文旅等商业类国企市场竞争力；坚持主业控股、辅业放开，进一步提升交通、供水集团等公益类国企服务效率和节本降支能力。以工投集团所属28户企业为重点，全面推开市属国企二级企业混合所有制改革，探索实施商业类国企骨干员工持股计划。继续推进国企市场化选聘职业经理人，改革市属国企负责人薪酬制度，不断完善现代企业运营管理机制。三是进一步强化财税管理。统筹财政预算资金，深化财政支出绩效考评，建立全面规范、公开透明的预算制度。创新财政投入模式，通过政府购买服务、政府与社会资本合作，吸引更多社会投资参与公共服务建设。继续加强地方政府债务管理，防范和化解债务风险。强化依法治税，切实提高税收服务水平。四是加快发展非公有制经济。落实省非公有制经济发展三年行动计划，全面开展民营经济发展改革试点，着力在扩大市场准入、完善服务体系、创新体制机制等方面取得更大实效。鼓励非公企业参与国有企业改革，重点在城市公共基础设施、新型城镇化、现代农业等领域推出一批投资项目。依托市中小企业公共服务平台，加快构建覆盖技术支持、创业辅导、融资担保、市场开拓全过程的服务体系，进一步创优非公经济发展环境。五是启动建设哈尔滨新区。作为十八届五中全会后首个获批的国家级新区，哈尔滨新区不仅是国家推动“一带一路”建设、加快新一轮东北地区等老工业基地振兴的重要举措，也为哈尔滨创新增动力、转型促升级提供了新的重大历史性机遇。我们要充分利用哈尔滨新区在深化改革上所具有的先行先试优势，围绕建成中俄全面合作重要承载区、东北地区新的经济增长极、老工业基地转型发展示范区和特色国际文化旅游聚集区，进一步加强顶层设计，积极推进体制机制创新，按照统筹建设、多规合一的原则，编制好新区规划和建设方案，细化配套相关政策，谋划建设一批基础性、先导性、支撑性重大项目，力争新区建设开好局、起好步。

（六）以对俄合作中心城市建设为牵动，着力拓展对外开放新优势新空间。坚持引进来、走出去相结合，更好地利用两个市场、两种资源，不断提高对外开放的层次和水平。一是加快建设对外物流大通道。联合南航、川航谋划开通北美货运航线，加密对俄航空货运航线，开通哈俄货运班列，依托北京-莫斯科国际列车、“辽满欧”通道开展对俄物流，以哈尔滨为起点打通至俄罗斯海参崴、布市、哈巴公路通道，使哈尔滨成为通过俄罗斯进入欧洲地区最快捷、最高效、成本最低的贸易枢纽。持续扩大对俄邮政小包裹出口量，确保跨境贸易电子商务国家试点城市建设取得更大进展。二是全力推进产业合作。抢抓我省申建对俄自贸区机遇，加快推动对俄贸易优化升级，积极引导组建大型外贸集团，支持企业深入拓展矿产资源开采、森林采伐、农业种植等领域合作。同步推进综保区功能区建设和招商引资，重点引进一批保税物流、国际商贸、加工制造等项目，年内实现封关运行。全力抓好临空经济区大飞机拆解、跨境电商物流基地、中国邮政（哈尔滨）航空邮件处理中心等项目建设，确保取得实质性进展。积极争取国家和省支持，力争中俄、中以合作产业园落户我市。三是努力扩大对外交流。重点加强文化、旅游、科技、金融、会展等多领域开放合作，积极开展油画、动漫等艺术品展览交易，高质量办好第六届“哈科会”，支持“中俄跨境金融合作联盟”开展人民币跨境结算等业务，积极培育和引进外语专业人才，为全方位扩大对外开放创造条件。

（七）更加注重改善民生，着力让人民群众更多更公平分享改革发展成果。今年重点实施10个方面、65个惠民行动项目，完成投资203亿元，切实兜住兜牢民生底线。一是改善居民住房条件。实行实物保障与货币补贴并举，全年开工建设保障房7296套、竣工11116套，为1.6万户住房困难家庭提供住房保障。改造农村危房1万户、60万平方米。持续提升物业管理水平，新增物业管理面积400万平方米，新创建物业管理示范项目50个，努力让百姓住得更舒心、更满意。二是建立更加公平更可持续的社会保障制度。继续推动各类社会保险提标扩面，城镇基本医疗保险纳入市级统筹，进一步扩大助保贷款覆盖面和医保异地结算机构数量。积极开展应对人口老龄化行动，深入推进养老服务业综合改革，鼓励社会力量建设养老社区，全年新增养老床位5000张。适时启动城乡低保动态调整机制，切实保障困难群众基本生活。三是推动教育改革发展。改善办学条件，创建义务教育标准化学校50所，新建中小学校20所，加固维修学校40所。深入推进集团化办学和教育云平台建设，实现区域内优质教育资源共享。妥善安置随迁子女平等接受义务教育，让更多困难家庭孩子享有公平发展的机会。建立民办学校奖补机制，鼓励社会力量和民间资本提供多样化教育服务。加快职业教育园区建设，抓好阿城、宾县两个省部共建农村职业教育改革，推动工学结合、产教融合、校企合作，努力为我市产业发展培育更多的适用型人才。四是提高城乡居民健康水平。启动市第一医院改革试点，扩大医院自主经营权，探索市属公立医院改革发展新路径。鼓励医生多点执业，促进优质医疗资源向基层、农村流动。完善公共卫生服务体系，免费为城乡居民提供12类45项基本公共卫生服务。落实全面两孩政策，促进人口均衡发展。着力建设食品安全城市，加快构建严密高效、社会共治的食品安全治理体系。五是大力推进精神文明和文体事业发展。扎实做好第五届全国文明城市创建工作，不断提高全市人民素质和社会文明程度。深入实施文化惠民工程，加快构建现代公共文化服务体系。积极开展“爱上交响乐”等高雅艺术普及活动，全力抓好哈尔滨大剧院、音乐厅运营管理，高水准办好第33届“哈夏”音乐会，持续扩大哈尔滨“音乐之都”品牌影响力和吸引力。办好哈尔滨解放70周年系列纪念活动。做好“731”、金上京申遗工作。继续抓好“百万青少年上冰雪”活动，高质量办好市第19届运动会，加快推进市奥体中心一期建设，着力打造城区10分钟健身圈，为丰富群众文体生活创造更多现实条件。六是持续打好精准脱贫攻坚战。坚持因地制宜、一村一策、一户一策，灵活运用发展生产、异地搬迁、生态补偿、教育培训、社保兜底等方式，确保完成年度贫困人口脱贫任务，为率先在全省实现全面脱贫打下坚实基础。七是提升社会治理能力。深入推进平安哈尔滨建设，完善反恐维稳、社会治安防控、公共安全管理和应急抢险处置体系，确保打造全国最具安全感省会城市取得更大实效。继续实施“强基工程”，进一步提升中心社区服务功能，新建农村示范社区36个。严格执行“党政同责、一岗双责、齐抓共管、失职追责”规定，及时排查消除各类安全隐患，坚决防止重特大安全生产事故发生。启动“七五”普法。积极构建多元化纠纷解决体系，完善动态管控、信息共享、协调联动机制，有效解决信访问题、化解社会矛盾。继续做好民族宗教、外事侨务、人防、统计、残疾人和国防教育及双拥、民兵预备役、档案、地方志等工作。

（八）全面加强自身建设，着力打造法治政府、创新政府、廉洁政府和服务型政府。巩固扩大“三严三实”专题教育成果，持续推进作风建设常态化、长效化，不断提高政府工作执行力、推动力和创造力。一是主动担当，真抓实干。担当实干是政府工作人员的职责所在、使命所系。面对经济新常态带来的重重压力和挑战，我们要始终保持一种振兴哈尔滨舍我其谁、时不我待的责任感和紧迫感，保持一种昂扬向上、奋发有为的创业激情和工作劲头，在产业发展、城市管理、改革开放、民生保障等事关全局和长远发展的重点工作上，主动作为、勇挑重担、攻坚克难、创新突破。大力弘扬踏石留印、抓铁有痕的务实作风，持续加大督办检查和责任追究力度，层层传导压力，环环紧扣落实，尤其对为官不为、庸政懒政等错误行为，一律公开曝光，坚决追责到底，让勇于担当、实干奉献真正成为广大公务人员的自觉行为和共同追求。二是践行宗旨，服务民生。越是在困难的时候，越是要重视保障和改善民生。我们要教育引导政府工作人员牢固树立全心全意为人民服务的公仆意识，进一步深入基层、贴近群众，着力解决好冬季供暖、物业服务、交通治理等百姓最关心、最直接、最现实的利益问题，真正做到权为民所用、利为民所谋。坚持言必行、行必果，确保各项惠民承诺不折不扣落实到位，决不给政府工作留下任何遗憾。三是依法行政，优化环境。把政府工作全面纳入法治轨道，自觉接受人大、政协和社会各界监督，注重听取工会、共青团、妇联等群团组织意见建议，切实做到依法决策、依法治理经济、依法协调和处理各种利益问题。进一步完善市民大厦服务功能，推进政务办公透明化、信息化、便捷化。始终保持行政问责高压态势，坚决查处吃拿卡要、失职渎职等违法违纪行为，让滥用权力、破坏经济发展环境的人和事在哈尔滨无藏身之处、无立锥之地。四是廉洁从政，干净干事。坚决把纪律和规矩挺在前面，自觉遵行廉洁自律准则，认真落实纪律处分条例，努力使广大公务人员正确用权、谨慎用权、干净用权。不断完善防腐治腐的制度体系，全面落实“两个责任”，严格执行“一岗双责、一案三查”，强化公共资金、公共资源、国有资产等领域的审计监督，从严惩处各类腐败行为，不断铲除滋生腐败的土壤，着力营造风清气正的发展氛围。

各位代表，2016年是本届政府履职的最后一年，也是全面建成小康社会决胜阶段的开局之年，做好今年工作责任重大、使命光荣。让我们更加紧密地团结在以习近平同志为总书记的党中央周围，在省委、省政府的正确领导下，按照市委的战略部署，紧紧依靠全市人民，万众一心、奋发图强、苦干实干，为开启哈尔滨全面振兴新征程、谱写人民美好生活新篇章而努力奋斗！
